# Primary mental healthcare for older people in India: between stigmatization and community orientation

**DOI:** 10.1007/s44192-023-00040-7

**Published:** 2023-07-28

**Authors:** Tom Kafczyk, Kerstin Hämel

**Affiliations:** School of Public Health, Department of Health Services Research and Nursing Science, Bielefeld University, Universitaetsstrasse 25, 33651 Bielefeld, Germany

**Keywords:** Health services for the aged, Mental health, Primary health care, Qualitative research, Community health services, India, Informal care

## Abstract

**Background:**

Questions of equitable access to primary mental healthcare (PMHC) for older persons in India have been examined mostly in terms of the coverage of services, although perceptions of mental health and old age and social norms at the community level should be considered in the shaping of PMHC approaches. The present qualitative study, therefore, examined how social perceptions and norms of mental health in old age are and should be considered in the design and implementation of primary healthcare approaches in India.

**Methods:**

A secondary thematic analysis of semi-structured interviews with key stakeholders (n = 14) of PMHC in India was conducted.

**Results:**

Four key themes emerged from the analysis, in which social perceptions and norms were discussed: (1) family participation and low threshold access to mental healthcare, (2) the position of community health workers as an important pillar of old age and mental health-sensitive community-based care, (3) the role of social cohesion and traditional values in fostering a positive and supportive community environment for old age mental health, and (4) the empowerment of communities, families and older persons through mental health education.

**Conclusions:**

PMHC, with its focus on mental health promotion, could be an important anchor for combatting negative attitudes about mental health and old age. The findings presented in this study can inform age-sensitive policies and programmes for mental health in India and could inform future research on the subject.

## Introduction and aim of the study

Mental health conditions are one of the leading causes of disability-adjusted life-years (DALYs) in low- and middle-income countries (LMICs) [1]. Consequently, mental health is increasingly recognized as a public health issue [2, 3]. Of all persons worldwide affected by mental health conditions, approximately 80% live in LMICs [2].

Older persons constitute a rapidly growing group that is especially vulnerable to poor mental health in LMICs [4]. Commonly, in the Indian public and social care system, people aged 60 or older are regarded as “old” [5]. While the phase of old age is characterised by diversity, older persons are more frequently affected by social, biological and psychological changes that can result in mental health conditions [6]. This holds true for India, a pluralistic country with a changing demographic and economic context, as well as a changing sociocultural context [7, 8]. By 2050, India is expected to have over 330 million people aged 60 or older, amounting to over 19% of the population [9]. According to India’s Census from 2011, most older persons live in rural areas (approximately 75%), are illiterate (approximately 73%) and live below the poverty line (approximately 66%) [10]. Mental health conditions *“[…] exist along a continuum from mild, time-limited distress to chronic, progressive, and severely disabling conditions”* [11, p. 1553] and affect well-being and emotions, behaviour, and cognition [12]. The leading mental health conditions in old age in India are cognitive impairments, depression, and anxiety, with higher prevalence rates reported for older persons residing in rural areas, older women, and socioeconomically disadvantaged older persons [13–15]. Mental health conditions such as late-life depression can occur in mild, moderate, or severe forms [16].

In India, primary mental healthcare (PMHC) based on the principle of community-oriented care is considered the most basic and viable foundation for addressing mental health among older age groups [14, 17]. Primary healthcare (PHC) allows for comprehensive mental healthcare close to the homes of older people to be strengthened. The broad spectrum of mental health promotion, including fostering a sense of positive mental health, mental illness treatment, rehabilitation and preventing relapses and recurrences of illnesses, is considered [18].

A recent policy analysis by Kafczyk and Hämel [19] demonstrated that older persons are increasingly recognized in legislation, strategies and programmes in India as a group that is vulnerable to impaired mental health; the policies themselves postulate community orientation as key to improving old age-inclusive PMHC. Across these policies, four strategies can be highlighted (for a detailed analysis, see Kafczyk and Hämel [19]):

1. *Supporting the family in a family-led approach*: The family is described as the largest resource in India in caring for older relatives who struggle with their mental health. However, the traditional family-based care system in India is described as breaking down, which has led to calls for reforming services for older persons [14, 20].
2. *Integrating community health workers (CHWs) into PMHC*: CHWs such as Accredited Social Health Activists (ASHAs) are discussed as support structures for families in caring for older persons with mental health illnesses [14, 20, 21], and policies include a vision that CHWs will take over tasks in old age and mental healthcare.
3. *Strengthening community empowerment and participation*: The significance of community empowerment for strengthening community participation in healthcare planning, implementation and monitoring is emphasized in policies in India. For example, as a mechanism for community health governance, Village Health Sanitation and Nutrition Committees were introduced through the National Health Mission, which conducts health planning, monitors government health and nutrition services and consists of community members, frontline health providers and locally elected representatives [22].
4. *Integrating traditional Ayurveda, Yoga and Naturopathy, Unani, Siddha, Sowa-Rigpa and Homeopathy (AYUSH) services into PMHC:* Much of the mental healthcare for older persons in India is already rendered in traditional care settings [23]. Against this background, policies highlight the integration of AYUSH as an opportunity for more comprehensive approaches to PMHC.

However, equitable access to old age-inclusive PMHC is limited [13], and this limitation is particularly pronounced in rural areas [14]. Questions of access and the development of ambitious, new policy approaches to better address mental health in old age are still being predominantly discussed as dependent on coverage-based factors, such as limited or absent mental health services at the community level and a shortage of trained mental health professionals [24, 25]. Current debates are less focused on the perceptions of mental health and old age at the family and community levels and the social norms that promote or hinder access to services [25].

The perception of mental health, especially the stigmatization of persons with mental health conditions in LMICs [26, 27] and in India, has already received attention in recent years [28–30]. In this context, the concept of mental health literacy, which *“[…] can be explained as the awareness and knowledge about mental health and its related problems that help in early recognition, management, as well as prevention […]”* has gained prominence [31, 32, p. 274, 33]. Occurring at the individual, societal and health system levels [26], stigma can be described as a problem of knowledge (ignorance), attitudes (prejudice) and behaviour (discrimination) [34].

However, the perception of old age or attitudes towards old age and the self-perception of ageing in terms of access to services in India have received comparatively less attention. With a still young population and high status differences, India was found to have a favourable view on the socioemotional aspects of ageing [35]; however, with increasing societal modernization and rising proportions of older persons in India, the view on ageing has become less favourable [35, 36]. Ageism, which involves stereotyping and discriminating against older people simply because they are old, is a widespread phenomenon in various societies [37–39], including India [40]. Perceptions of both mental health and old age can be understood as manifested in social norms. Social norms are the *“[…] rules and standards that are understood by members of a group, and that guide and/or constrain social behavior without the force of laws”* [41, p. 152].

The influence of perceptions and social norms can be expressed subtly, with families in India and older persons themselves not knowing when they or a family member is suffering from a mental health impairment [13]; in India, poor mental health in old age is often disregarded as a normal part of ageing [14, 21, 25, 42]. This may also occur more directly when family members and community members regard older persons’ difficulties with their mental health as being used to seek attention [42]. These perceptions and behaviours can be seen within the framework of stigmatization and discrimination because of mental health and age. Such misconceptions and forms of stigmatization and discrimination can also be found among healthcare workers [43, 44]. For example, misconceptions among CHWs could lead to an under-identification of mental health conditions within the old-age population in India [20, 45]. As a result, older people’s access to dignified healthcare is limited, and they are deprived of their rights [46]. Moreover, inequities in access to health services are particularly manifested at the intersection of old age, female gender, and low socioeconomic status (SES), with, for instance, Srivastava et al. [47] showing that the oldest old-age groups, females, and persons with low economic and social statuses (e.g., scheduled tribes) have a lower propensity to seek care for mental health illnesses.

## Methods

### Aim of the study

In this study, we analyse how social perceptions and norms of mental health in old age are considered in the design and implementation of primary healthcare approaches at the federal level in India. To sharpen the analysis, we first want to understand how social perceptions and norms of mental health in old age facilitate or hamper access to care. We focus on family- and community-based care, as these approaches are important pillars and at the centre of the current political agenda in India. By identifying leverage points, we aim to provide findings that can guide further research and the design of policies within PMHC in India and other LMICs.

### Study design

This study is part of a larger research project. Its key objective is to explore and understand how the evolving public health system in India addresses the mental health needs of older people. The focus is on the emerging policy field that shapes PMHC for older people in India. Specific challenges and opportunities for strengthening age-inclusive PMHC in India are analysed in more detail. The research project provides guidance and recommendations for real-world improvements. A mixed-methods approach was used, comprising a policy document analysis [17, 19] and semi-structured interviews with key stakeholders (Kafczyk and Hämel, in review).

The study presented herein was designed as a secondary analysis [48–50] drawing on expert interviews with federal-level stakeholders in the policy field of PMHC in India. In the semi-structured interviews, we did not explicitly ask about social norms and perceptions of mental health and old age or their relevance to old age-inclusive PMHC. However, all interviewees reflected on these topics, and we focused on them through follow-up questions. Consequently, we performed a supplementary analysis [48–50] to advance our understanding of the challenges to and opportunities for PMHC for older persons in India. Because we are revisiting our own data, we see additional benefits in terms of objectivity due to the emotional distance from the material [51].

The consolidated criteria for reporting qualitative research (COREQ) were followed [52]. In addition, we followed the recommendations of Ruggiano and Perry [48] with regard to performing secondary data analysis to increase the rigor of the overall research project. Both the first and secondary analyses were conducted by the same researchers, and similar analytical techniques were used.

### Sampling

Stakeholders (experts) responsible for the development, implementation or control of solutions, strategies, or policies at federal-level and who have privileged access to policy-level information on a national level [53, 54] pertaining to mental healthcare for older persons were sampled for this study. The sampling was based on purposive sampling. Potential interview partners were identified by a review of the literature, including policy documents, conference papers and a web search. In line with the interdisciplinary perspective approach [55] that was adopted, experts from all relevant political and stakeholder groups were sampled. The experts were selected based on the following criteria: (1) being a leading expert, (2) over 10 years of work experience, (3) involved in federal-level policy processes, and (3) having good command of English. Potential participants were invited via e-mail, telephone or occasionally in face-to-face meetings with the first author. In all cases, they were provided with information about both the study and their participation. Written informed consent was obtained from all participants that included the use of their data for this secondary analysis. Out of the 34 persons who were contacted, a total of 14 expert stakeholders participated. Most persons who did not participate did not reply to our invitation, while some declined to participate because they either felt insufficiently knowledgeable or were on sick leave. Recruiting of further participants was stopped as data saturation [56] emerged around the arising themes [56]. Five experts were female, nine were male. They were representatives of government advisory agencies (n = 3), representatives from research institutes (n = 2), clinicians and representatives of professional associations (n = 5), civil society representatives (n = 3) and patient representatives (n = 1). The inclusion of civil society and patient representatives was important, as both represent and advocate for older persons.

### Data collection methods

The interviews were conducted depending on the participants’ preferences, i.e., either in the participants’ office (n = 10) or in a quiet public place (n = 4). Other than the principal investigator and the person being interviewed, no one else was present during the interviews.

Interviews were conducted in English as this is a common language among experts with federal-level influence in India. Challenges arising from employing a translator or using translations were avoided in this way [57]. Since none of the interviews were difficult to conduct in terms of language, we also saw no concerns that subtleties or nuances could be lost due to the interviewee and the interviewer not using their first language. Overall, however, we encourage future studies on the subject to also include non-English-speaking experts to broaden the sample to non-English-speakers and stakeholders from regional and local contexts.

We are cautious in our interpretations of the data because of potential variations in the sociocultural-political context from the time of data collection, i.e., in 2017 and 2018, to the current climate in 2023. Nevertheless, since social norms and perceptions of mental health and old age change slowly [58] and few developments in the mental healthcare system have occurred due to the general standstill caused by the new coronavirus disease (COVID-19) pandemic [59], the data are interpreted as being highly relevant. However, the digitalisation of the health system has been strengthened since the COVID-19 pandemic [60], and these developments are not reflected in our data.

An interview guide with a set of predetermined open-ended questions was developed prior to conducting the research project to allow for flexibility within the interviews (i.e., to ask questions that emerge during the dialogue). Especially in expert interviews, the form of open interviews is postulated to be able to capture the expert’s views and opinions and her or his own interpretation of reality [54]. At the beginning of each interview, we asked the participants about their sociodemographic and professional background to inform the data analysis; for reasons of anonymity, this information is not presented here. The interview guide comprised questions on central access challenges and opportunities for strengthening mental healthcare for older persons at the community level. Furthermore, we asked about mental healthcare approaches at the PHC level and how these address central challenges and opportunities. In line with our understanding that mental health conditions exist along a continuum ranging from mild to severe [11] and that the PHC level offers a spectrum of care from mental health promotion to the prevention of relapses and recurrences of mental illnesses [18], the questions were not limited to either the severity of mental health conditions or the type of care.

The interviews (median length of 50 min, with an interquartile range of 26 min) were recorded and transcribed verbatim. The principal investigator conducted all interviews. In addition, field notes and a research diary were kept.

### Data analysis

For this study, thematic analysis was used [61]. Since social norms and perceptions of mental health and old age were discussed in all of the interviews, we deemed it valid to purposively select all interview transcripts to re-examine the data set to explore the specific research questions in this study. All sections in the transcripts that referred to perceptions and social norms were selected for data analysis. The first author conducted inductive coding, in which relevant transcript sections were read and pertinent passages were coded with MAXQDA 2022 [62]. From the first inductive codes, the data were condensed and categorized by the first author in an iterative process until an increasing number of patterns emerged. The consequent development of final themes was discussed and reviewed by the research team until no new discrepancies emerged and consensus was achieved [63]. In a last step, a detailed analysis was conducted in which the themes were comprehensively described. This detailed analysis has repeatedly been discussed and further developed by the research team.

Four main categories or themes around which the thematic structure was built emerged in the analysis: (1) family participation and low threshold access to mental healthcare; (2) the position of community health workers as an important pillar of old age and mental health-sensitive community-based care; (3) the role of social cohesion and traditional values in fostering a positive and supportive community environment for old age mental health; and (4) the empowerment of communities, families and older persons through mental health education.

### Ethical considerations

Ethical clearance for both the initial and this supplementary analysis was obtained from the Institutional Ethics Committee, Bielefeld University, Germany (no. 2017074).

## Results

### Family participation and low threshold access to mental healthcare

The experts stress the relevance of families in mental healthcare for older persons. Often, relatives take—or do not—older persons to health services; as one expert stated, *“If you can control that family level, you do not need any other level”*^1^ (E4, Pos. 62). However, a widespread problem at the societal and family levels involves a lack of knowledge of mental health that is compounded in old age and a “mental health stigma”.

*“Mental health is not considered a disease state in our culture, and nobody wants to discuss their stress or depression or memory failure. A lot of stigma is attached to it. And if you look at conditions like schizophrenia or manic depressive psychosis, these are actually hidden and never discussed.”* (E9, Pos. 21)

The situation is even more serious in rural areas.

*“I have worked a lot in the villages and in rural areas with older persons. […] We were not able to find anybody who can give us questions on the mental health of elderly people. Therefore, the knowledge and awareness were completely missing. When you say mental health, a lay man says, ‘Oh am I mad’? It is only for crazy people. Therefore, the lack of awareness was very palpable.”* (E11, Pos. 13)

Furthermore, the interviewees describe declining attitudes towards older persons from their families, which they see as particularly problematic when older persons become “dependent” on other family members in everyday life. The respected and dignified perception of older persons that was held together by their control of family decisions and assets is eroding. Instead:

*“An elderly person, it may sound very cruel, but he is not a very productive member of the family. So I rather spend my money and my resources on my child than on this man. It happens, I am not saying it is a general rule, but it can happen suddenly and it can happen broadly.”* (E7, Pos. 31)

Against this background, families often fail to support and care for older family members who struggle with their mental health. Indeed, the opposite can occur: families can contribute negatively to the mental health of older family members. Experts indicate that forms of abuse and mistreatment—because of mental health conditions—can happen.

*“Families have no idea [of dementia, added by the authors]; that is why they mistreat, they shout, they throw people out of their homes.”* (E11, Pos. 113)

Because of this lack of knowledge, it is challenging for family caregivers to recognize mental health impairments and to know what to do in these cases. Furthermore, experts describe a lack of openness among older people and their families to talk about mental health.

*“If am a depressed elderly person, say 80 or 70, I am depressed and I chose to sink in that depression and not discuss it with family members. Or the family members do not discuss it with me. And if I chose to ignore it or I chose to see it as a part of normal ageing, then I will not even seek medical help.”* (E3, Pos. 31)

Consequently, health-seeking behaviour outside of the family is often challenged, or people may *“[…] delay health seeking until often the situation is very advanced […]”* (E2, Pos. 55)*.* Furthermore, families are described as being afraid of their reputation and position in society and therefore are reluctant to seek help.

*“Who in my family will take me for a disease that does not have manifest symptoms? Nobody will.”* (E7, Pos. 39)

This holds especially true for the utilization of psychiatrists where experts see a stronger avoidance because of the stigma attached to specialized mental healthcare. To overcome this barrier, experts advocate for strengthening the competences of PHC physicians to allow for low-threshold access to mental healthcare.

However, the interviewees highlight that challenges in mental healthcare in the family setting cannot be understood solely as issues of knowledge or stigmatization. Many older people belong to families from a poorer SES background in which neither health nor—especially—mental health is a priority since basic needs are often not met.

*“[…] if they recognize it [mental health disease, added by the authors] as an ailment they [the family, added by the authors] tend to underplay it because there are many more physical existential issues in these families than looking after your mind.”* (E3, Pos. 31)

This should be understood as important, as populations in rural areas and from lower SES backgrounds in particular—the traditional focus groups of PHC in India—lack awareness and knowledge of (old age) mental health.

Against this background, financial security is regarded as important and discussed in terms of social protection, pensions, and financial compensation for family caregivers. According to the interviewed experts, while this debate is not drawing as much attention as it should in India, it could be an anchor point for strengthening the role of families in mental healthcare for older persons and to raise the position of older persons in their families and communities.

### The position of community health workers as an important pillar of old age and mental health-sensitive community-based care

In the interviews, community-based health services are addressed as an important pillar of PMHC for older persons. In particular, outreach by community health workers (CHWs), such as ASHAs or Anganwadi Workers, is prominently promoted. They are discussed as being able to (1) provide mental healthcare to affected or vulnerable older persons close to their homes (e.g., simple forms of therapy through outreach), (2) function as coordinators of health services and connectors (*“connecting link between the community and health services”,* E13, Pos. 54) and (3) train and sensitize families and support them in their caregiving roles.

However, these roles are difficult to establish in practice due to several hindering factors that are highlighted by the experts. These include the CHWs not being properly trained in old age mental health and being prone to downplay mental health conditions in old age; for instance, they may believe that mental illnesses cannot be treated. The interviewees acknowledge the need to provide basic training in geriatric and mental healthcare to CHWs. One important training component for CHWs should be *“[…] that this could be treated that it’s a treatable condition.”* (E12, Pos. 49). In addition, these workers need to be trained on the following:

*“How can people dealing with mental health problems be identified? What kind of broad classifications are there? What kind of help is available? How can people be sent to those services? How can people get back in the community? How should follow-up visits be paid?”* (E13, Pos. 62)

Nevertheless, a major barrier to CHWs playing a prominent role in outreach services to older persons with mental health conditions is the attached stigma.

*“Some mental health problems are kept under the cover and are ignored by people, and they don’t approach professionals. Professionals reaching out to them becomes very difficult. […] So this is an issue for which you have to work and have policy and programs and more outreach and make it more visible and have more services available at their doorstep.”* (E14, Pos. 15)

Furthermore, a new direction for the tasks of CHWs would contrast with the current focus on maternal and childcare, immunization, and neo-natal and ante-natal care. Experts warn that CHWs already have limited capacities and time to take on additional tasks. In this context, the experts suggest creating a new cadre of basic old age care providers trained in mental health. There have been initiatives, mostly from private agencies, that offer, for instance, geriatric home care services, but there is no programme on a national basis that could be developed.

Experts also encourage policy- and decision-makers to develop a new system of incentivizing CHWs. The current system does not incentivize noncommunicable disease care, particularly mental health management, which can negatively impact the motivation of CHWs to work on mental health.

*“[…] the ASHA payment it’s an incentive driven payment; it’s not a salary driven. So if pregnancy is registered or immunization is given it’s countable. What is the count of a depressed person feeling happy? […] There is no count. Therefore, there is no incentive.”* (E9, Pos. 55)

For changes in the work package of CHWs, experts emphasize the importance of political will. The experts understand that policy-makers do not currently perceive mental health and old age care as priorities.

### The role of social cohesion and traditional values in fostering a positive and supportive community environment for old age mental health

For most experts, the community is understood as an important anchor and resource in India for supporting the mental health of older persons that should be an integral part of any PMHC approach. The experts regard the community as a resource for fostering a positive community environment for the mental health of older persons. A pronounced theme is a sense of social cohesion and support, which they see as more distinctive in rural areas, as they are connected to a traditional understanding and values of community in India.

*“[…] they have their traditional structures; they are still very strong. […] They [older persons, added by the authors] sit under a tree and then they talk for hours; they probably play cards; they probably play some local game. Thus, the bonding is very strong.”* (E7, Pos. 95)

For older persons, traditional activities such as praying, singing, or meditating contribute to a sense of social cohesion and to their positive mental health. Experts see a trend of more self-organized community groups—with and for—older persons that appear to provide mutual support and a platform to advocate for different topics and needs, such as counselling for older persons. Often, they do activities together, such as physical exercises, laughter therapy or yoga.

*“Usually what is happening is that a lot of senior citizen associations have come up in various parts of the country. With their presence and with their activities, they are also focusing on the needs of older people. […] Or they are trying to provide certain kinds of recreational facilities which provide some kind of activities for people affected by different kinds of needs.”* (E14, Pos. 45)

However, the interviewees are also concerned about a slow but steady breakdown of traditional community values and structures. This trend can be seen in rural areas and is already more advanced in urban areas, where older persons are more isolated.

Therefore, according to the experts, social cohesion, bonding and organized groups with and for older persons, whether in rural or urban areas, should be strengthened and facilitated by the state, integrating community volunteers into approaches to strengthen community ownership. In this regard, the interviewees perceive local governance structures (e.g., village panchayats) as a potential resource for ensuring that older persons have access to adequate community-based structures.

### The empowerment of communities, families, and older persons through mental health education

Experts emphasize that a lack of awareness of the different forms of mental health conditions in old age and stigmatization are decisive hindering factors in unfolding a stronger role of families and communities in old age mental healthcare. Old age mental health education is described by experts as a critical starting point to enable older persons, their families and communities to understand *“[…] how to manage, how to cope and how to live with it [mental health issues, added by the authors]” and how to “age well”* (E14, Pos. 53).

*“[…] creating awareness is very important; creating awareness and destigmatizing mental health challenges is very important. Unless I come out and tell you my problem, how will you solve it, you know.”* (E7, Pos. 95)

The experts perceive progress as a reason to be optimistic. Initial steps have been taken in raising the awareness and knowledge of old age mental health in Indian society, including among the family, as *“[…] family has become somehow aware that mental health is an issue”* (E9, Pos. 63), and among health workers at the community level. Different reasons are given for this upwards trend. One reason is the growing number of older persons and increased recognition of noncommunicable diseases in India. A growing number of private not-for-profit organizations are also working on this topic. In addition, there

*“[…] is now a lot more counselling available through the internet also. This is available through senior citizens associations, through media, through more availability of geriatric services, which is making older people aware of the changes that are happening in their body, whether it is related to insomnia, whether it is related to stress, whether it is related to behavioural problems or anxiety, whether it is related to counselling, or whether it is related to other memory problems […].”* (E14, Pos. 53)

Although the experts expect that this change in perceptions, knowledge and behaviour will be a slow process because *“[t]hat is a cultural thing”* (E7, Pos. 63), they see connecting points. It is very important to educate family caregivers. Specifically, the experts promote the idea of a national training programme for family members.

*“ […] the caretaker or care provider also has to be taken care of in terms of dealing with some of the mental health concerns [of older persons, added by the authors].”* (E14, Pos. 27)

Old age mental health education should be anchored at the PHC level. The experts strongly promote concerted efforts to integrate important stakeholders such as administrative units at the community level, CHWs, health professionals and younger age groups. In particular, the integration of CHWs is seen as important for destigmatizing mental health and educating communities and families about mental health in general and specifically in old age. To promote positive attitudes towards older persons, it is important to highlight that

*"[t]hey are contributors to society. To families, a healthy, mentally fit older person is an asset. To their family, to their community, to society, they can be good caregivers. Therefore, a healthy mentally fit older person can contribute a lot to the development process of the country and to the family.”* (E14, Pos. 55)

In this regard, however, the experts warn that raising awareness and encouraging positive mental health-seeking behaviour need to go hand in hand with the availability and development of age-appropriate mental health services.

## Discussion

In view of the accelerating demographic transition in India, the mental health of older persons has gained increasing attention in public health discourses. Comprehensive and community-based primary mental healthcare (PMHC) is postulated in policies, but a high treatment gap thereto persists. Many factors play a role in shaping equitable access to PMHC. In our study, from the perspective of key stakeholders, we focused on (1) how social norms and perceptions of mental health in old age at the family and community levels are seen as facilitating or hampering factors for old age-inclusive PMHC, and (2) how this should affect or has affected the concrete design and implementation of care approaches. We focused on family- and community-based care, as these approaches are at the centre of current political approaches. From an integrated perspective, we present the main conclusions in the following sections.

### Stigmatization and low old age mental health literacy hamper access to age-inclusive PMHC

According to the findings of our study, the widespread stigmatization of mental health conditions in old age is a persistent part of Indian culture and a significant barrier to PMHC. In particular, older persons in rural areas and with low SES seem to be affected. This is closely associated with a lack of awareness and knowledge of the facets of mental health in old age at the family and community levels, which suggests low old age mental health literacy. For instance, earlier studies highlight that traditional and supernatural disease explanations for mental health conditions are common in India [25, 64, 65]. Our study confirms that negative perceptions of mental health *and* of old age potentially aggravate each other. When people are getting older, there is a greater danger that negative stereotypes are internalized, which can affect functioning and health [39]. For instance, Levy et al. [66] show a relationship between holding negative age stereotypes and the development of Alzheimer’s disease.

Stakeholders continue to see the family as playing a prominent role in mental healthcare for older persons in India, although they highlight a slow erosion of traditional family care systems and underlining norms in India [14, 67]. This is in line with the current familialistic policy approaches in India [19]. Nevertheless, the widespread low mental health literacy of caregivers and declining attitudes towards older persons in the family setting make the feasibility of this approach questionable. Similarly, as Patel et al. [13] observe in Jodhpur, families and older persons themselves do not know when either they are or a family member is suffering from a mental health impairment. The caregiving role of family members is further hampered by the small amount of (financial) support they receive, which is reminiscent of findings from other LMICs [68]. Especially among families from a low SES background, mental health is described as not being a priority compared to more basic needs. These contexts constrain appropriate societal and familial behaviour towards old age mental health and support-seeking behaviour. According to the results of our study, there is a widespread reluctance to speak openly about the mental health of older persons; this includes not only family members and the local community but also the PHC workforce. Consequently, access to PMHC is limited, and often only severe cases end up in the health system.

### Integrated family- and community-based interventions to foster a positive understanding and practice of old age mental health(care)

The importance of PMHC is highlighted in our study, but the challenges in reaching older persons with or at risk of mental health conditions need to be acknowledged so that they can be better addressed. The PMHC system and the policies thereof should emphasize community-oriented mental health promotion and prevention and self-help oriented support and offer low-threshold access to mental health services. The experts see a wide-ranging need for intersectoral old age mental health education, as an important component of mental health promotion, anchored at the PHC level, to improve old age mental health literacy, from the individual, family, and community level to that of healthcare workers. Although slow progress can be expected, this study suggests that a positive understanding of mental health *and* of old age can be fostered. This would help to create a positive environment in which the older persons who are affected, their families, communities and the healthcare system can interact openly and be informed about mental health, ultimately improving dignified access to old age-inclusive PMHC [46]. The willingness to seek professional help for mental health conditions is already a strong predictor of actually seeking help [69] and is positively influenced by community mental health awareness campaigns [70].

Shifting tasks from specialist mental health services to lay counsellors or low-intensity therapy methods as a form of increasing accessibility to collaborative care has received more attention in LMICs and in India in old age mental healthcare [19, 44, 71] and is supported by findings in this study. CHWs should be promoted as key providers of community-based mental healthcare—including in home-based care—and actors in destigmatizing mental health as well as trainers and supporters of family caregivers. As has been observed in other LMICs [68], we found that family caregivers in India need adequate support so that they can fulfil their socioemotional and mental health support role for older family members without compromising their own health. It is unquestionable that families with more knowledge about mental health have the necessary information to provide better care for older family members [25]. In a study by Scazufca et al. [72] from Brazil, low-intensity psychosocial and psychoeducation interventions delivered and led by CHWs partly in the homes of older persons were shown to be effective in recovery from old-age depression and in the reduction of barriers to care. Similar results and care models have also been shown to be effective in India [73–75] and are promoted to effectively improve care for mental health conditions in old age [76]. However, according to the results of our study, transforming the scope of practice of CHWs in India to include mental healthcare for older persons is a major challenge. The focus of CHWs is still on maternal, child, neo-natal, and ante-natal care and infectious disease prevention. Moreover, CHWs have only limited capacity. Because of the special requirements of old age mental healthcare and the challenges that are involved in transforming the scope of existing cadres of CHWs, some experts suggest developing a new cadre of CHWs that care for older persons, including their mental health. This cadre could involve persons from communities with lived experiences of mental health conditions [77].

In this study, community-based organized groups of older persons appear to be a promising model in which older persons support themselves (peer support) and advocate for their issues in collaboration with local governance structures. These groups help to strengthen social engagement and can reduce isolation and loneliness [78]. A recent systematic review by Makhmud, Thornicroft and Gronholm [27] shows the importance of (indirect) social contact interventions in reducing mental health-related stigmatization. These interventions encourage contact, either in person or through other means, between the stigmatized group and those displaying stigmatization; although it is not clear how these work in the case of old age, such campaigns have been successfully used in India to reduce mental health stigma in the general population [29, 79]. For future policies, greater consideration could be given to approaches that foster positive community environments and social cohesion as means to improve mental health promotion.

Considering the influence of local contexts [76, 80], we can assume regional variations in social norms and perceptions of mental health and old age on a community level. Further research could examine the heterogeneity of Indian regions. In any case, mental healthcare policies and practices should take context and culture-specific factors into account [81]. Furthermore, age-specific services should not be developed in parallel with already existing structures; rather, they should be an integral part of a holistic, community and needs-oriented approach [19]. In this sense, a strength of PHC is that it develops approaches for the whole population independent of age. Nevertheless, we have been able to show that it is important to develop age-appropriate care approaches particularly in view of the demographic shift in India. PHC offers a range of opportunities including to integrate social aspects of care and mental health promotion that starts as early as possible in the life of a person.

## Limitations

By interviewing a multistakeholder sample, we were able to provide a broad overview of social norms and perceptions of mental health and old age on a community level that affect mental healthcare for older persons. Experts from civil society and patient representative organization were interviewed in their role as advocates for older persons affected by mental health conditions, although this role suggested their limitation as being simultaneous service providers. However, this does not replace the inclusion of the target group itself. Further research needs to be conducted to analyse the perspectives of older persons with and without lived experiences with mental health conditions, their families, communities, and healthcare workers and to consider the findings in the development of the PMHC system. This approach would also help to broaden our understanding of the barriers and facilitating factors to age-inclusive PMHC comprising of the knowledge of older persons of available mental healthcare resources.

In this study, we have implicitly focused on the traditional normative family structure of extended family households that also underpin policies [19]; with that being said, new varieties of family forms in India [67], such as older persons living alone, have not been discussed explicitly in this study; future studies can therefore expand their scope and address different family forms, including older persons without social (familial) support structures.

## Conclusion

Given the population ageing in India, the perceptions of and attitudes towards mental health and old age need to be considered to improve the equitable access to and participation of older persons and their families in PMHC. The stigmatization of mental health—especially in old age—is a major barrier that is deeply rooted within the family and community care system. While the families and communities of older persons are foreseen as playing a strong role in PMHC in the Indian public health system, supporting them through information and knowledge as well as relief services is an important prerequisite to making them active participants. The increasing disintegration of family support systems in rural and urban areas necessitates a more prominent role for outreach support provided by community health workers. The PMHC, with its focus on mental health promotion, could be an important anchor for combatting negative attitudes about mental health *and* old age, especially the stigmatization of mental health and ageism. The findings presented in this study can inform age-sensitive policies and programmes for mental health in India and other LMICs and could inform future research on the subject.

## Data Availability

The data sets analysed during the current study are not publicly available because they consist of interview data, for which confidentiality cannot be safeguarded. Reasonable requests to access the data should be directed to the corresponding author.

## Abbreviations

ASHA: Accredited Social Health Activist
AYUSH: Ayurveda, Yoga and Naturopathy, Unani, Siddha, Sowa-Rigpa and Homeopathy
CHW: Community health worker
LMICs: Low- and middle-income countries
PHC: Primary healthcare
PMHC: Primary mental healthcare
SES: Socioeconomic status

## Footnotes

1 Direct quotes from interviews have been linguistically edited to improve readability.
